# Supplementary material for: Old and New Biomarkers for Infection, Inflammation, and Autoimmunity in Treatment-Resistant Affective and Schizophrenic Spectrum Disorders
Source: Pharmaceuticals (Basel). 2022 Feb 28;15(3):299. doi: 10.3390/ph15030299 (PMC8949012; doi:10.3390/ph15030299)
Supplement: Supplementary file 1 [file pharmaceuticals-15-00299-s001.zip › pharmaceuticals-1580583-supplementary.pdf]

pharmaceuticals

MDPI

Supplement

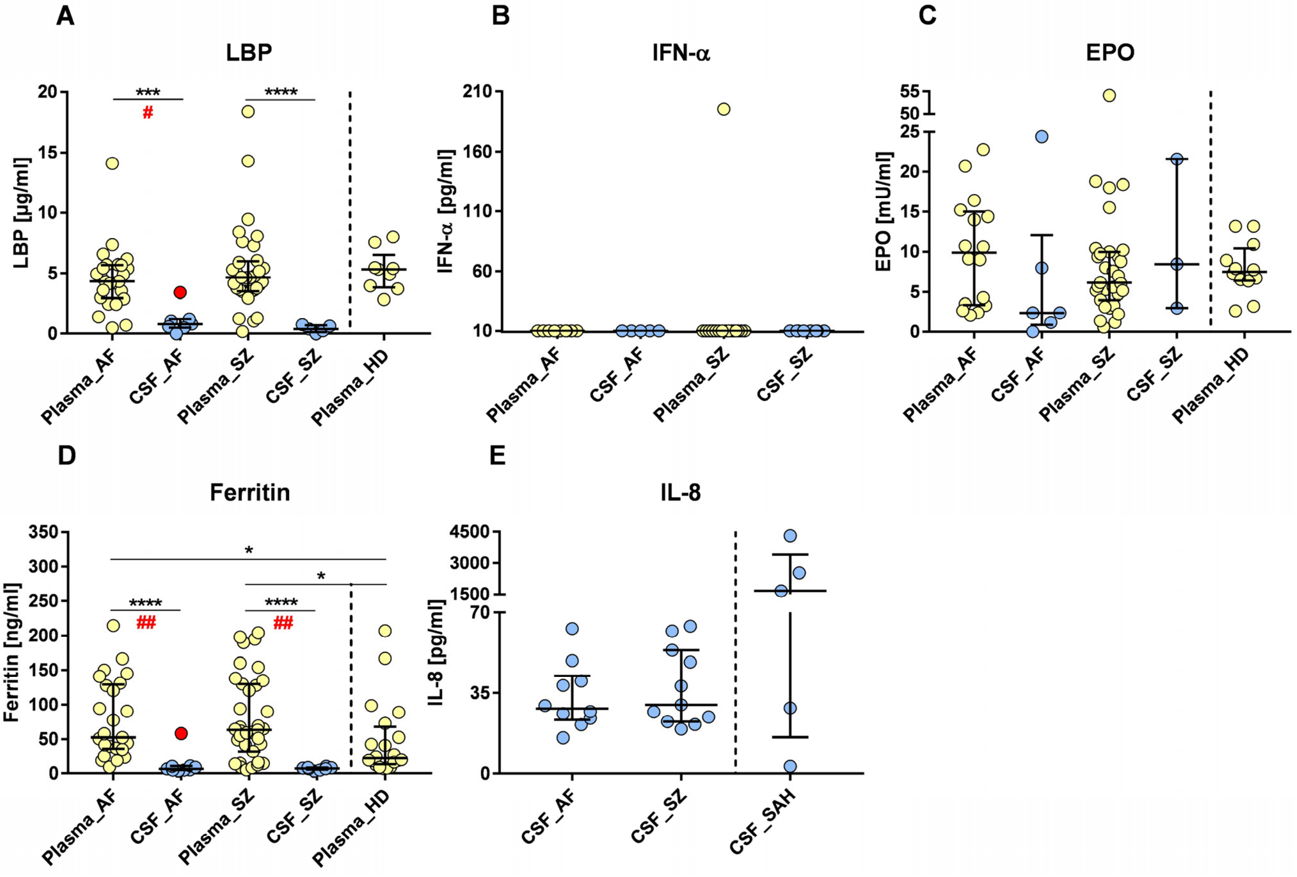

Figure S1: Biomarker concentrations in plasma and CSF of patients with AF, SZ, SAH, and HDs. Concentrations of LBP (**A**), IFN-α (**B**), EPO (**C**), ferritin (**D**), and IL-8 (**E**, CSF only) are shown as scatter plots, with bars indicating the medians, including interquartile ranges. A selected outlier patient in the CSF_AF group is color labeled (red symbol) in **A** and **D**. Wilcoxon rank-sum test was performed to analyze differences among groups, with significant differences being marked by $^{*}p \leq 0.05$, $^{***}p \leq 0.001$, and $^{****}p \leq 0.0001$. Wilcoxon matched pairs signed-rank test was performed to analyze differences between patient-matched pairs (plasma vs. CSF), with significant differences being marked by # $p \leq 0.05$, ## $p \leq 0.01$.

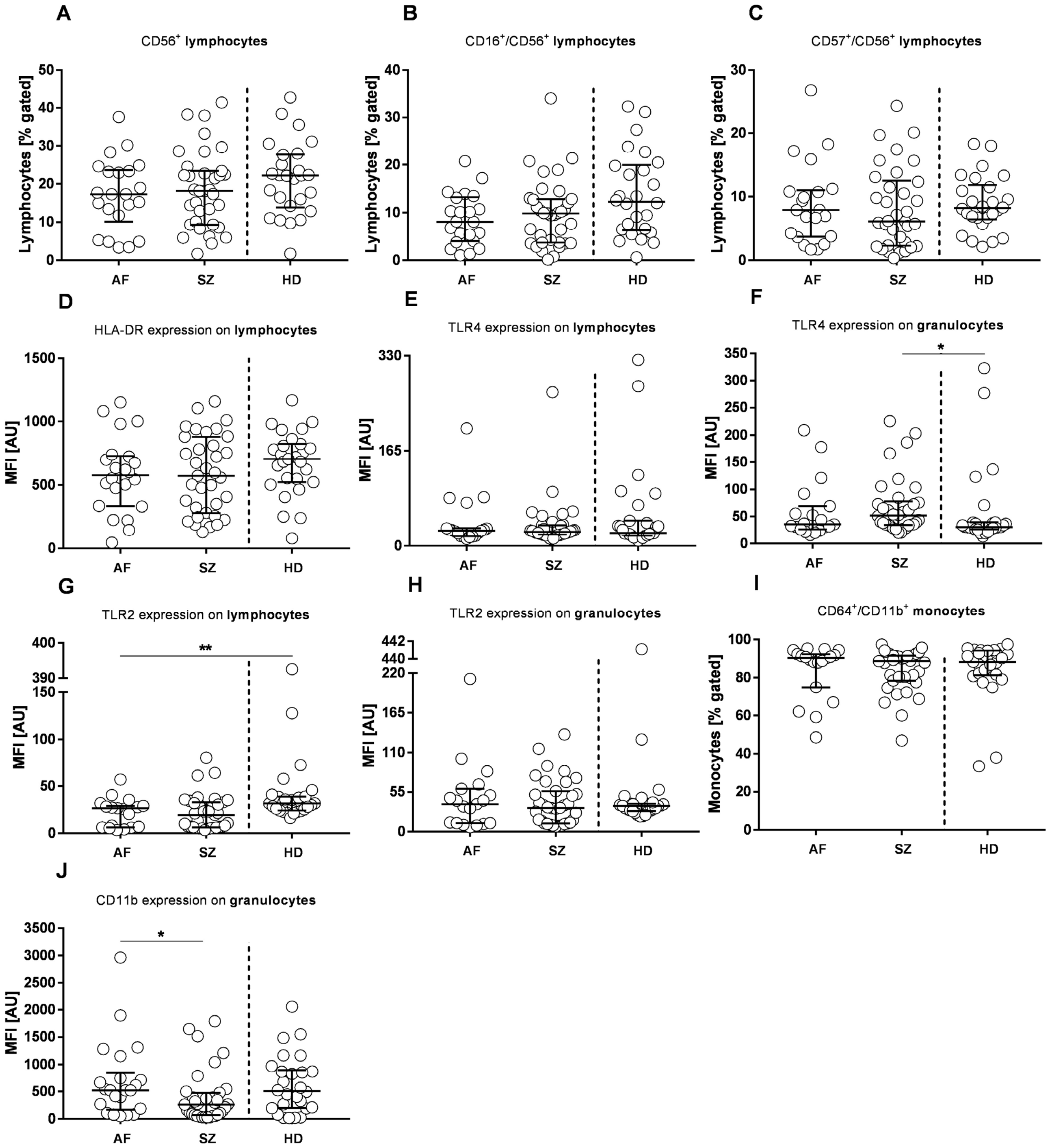

Figure S2: Flow cytometric profiles in patients with AF, SZ, and HDs. Relative amounts (% gated) of $CD56^+$ lymphocytes (**A**), $CD16^+/CD56^+$ lymphocytes (**B**), $CD57^+/CD56^+$ lymphocytes (**C**) and $CD64^+/CD11b^+$ monocytes (**I**) are shown. Lymphocyte expression densities of HLA-DR (**D**), TLR4 (**E**) and TLR2 (**G**) are shown. Granulocyte expression densities of TLR4 (**F**), TLR2 (**H**) and CD11b (**J**) are shown. Expression densities are given as arbitrary units (AU). Bars indicate the respective medians, including interquartile ranges. Wilcoxon rank-sum test was performed to analyze differences among groups, with significant differences being marked by $^*p \leq 0.05$, and $^{**}p \leq 0.01$.

Table S1: Correlation values for selected plasma cytokine concentrations in patients with affective (AF) and schizophrenic (SZ) spectrum disorders, and healthy donors (HDs). r indicates the spearman correlation coefficient. Statistically significant p values (p≤0.05) are highlighted. Notable is the strong plasma level correlation between IL-1β and TNF-α in HDs, which also represents the highest correlating pair of cytokines in SZ patients, but shows a much weaker correlation in the AF patients. Other significantly correlating pairs of plasma cytokine levels in the SZ group are IL-8 vs. TNF-α, IL-8 vs. IL-1β, IL-8 vs. IL-10, and IL-1β vs. IL-10, all of them showing both, lower and not significant, correlations in AF patients. In the latter, the highest correlation is found between IL-10 vs. TNF-α, however not being different in the SZ group, and a trendwise negative correlation in HDs (p=0.78).

	AF			SZ			HDs		
	**Size**	**r**	**P value**	**Size**	**r**	**P value**	**Size**	**r**	**P value**
**TNF-α vs. sCD25**	23	-0.07	0.74	34	0.08	0.66	19	-0.36	0.13
**IL-8 vs. sCD25**	25	-0.17	0.40	34	-0.09	0.63	17	0.11	0.67
**IL-6 vs. sCD25**	23	0.22	0.32	29	-0.16	0.42	17	0.37	0.14
**IL-1β vs. sCD25**	26	-0.30	0.14	34	0.21	0.22	15	-0.09	0.75
**IL-10 vs. sCD25**	25	0.08	0.71	35	-0.08	0.63	18	-0.06	0.80
**IL-8 vs. TNF-α**	22	-0.32	0.15	34	0.49	**≤0.01**	17	0.37	0.14
**IL-6 vs. TNF-α**	20	0.12	0.61	30	0.22	0.25	17	-0.12	0.64
**IL-1β vs. TNF-α**	23	0.06	0.77	34	0.62	**≤0.0001**	17	0.75	**≤0.001**
**IL-10 vs. TNF-α**	22	0.53	**≤0.05**	35	0.48	**≤0.01**	20	-0.07	0.78
**IL-6 vs. IL-8**	23	-0.23	0.28	30	-0.04	0.83	15	0.04	0.89
**IL-1β vs. IL-8**	25	-0.03	0.90	37	0.39	**≤0.05**	13	0.36	0.23
**IL-10 vs. IL-8**	25	-0.20	0.34	35	0.43	**≤0.01**	16	-0.02	0.94
**IL-1β vs. IL-6**	23	0.26	0.23	30	0.32	0.08	15	-0.13	0.64
**IL-10 vs. IL-6**	23	-0.11	0.63	30	-0.04	0.82	16	0.42	0.10
**IL-10 vs. IL-1β**	25	-0.27	0.19	35	0.43	**≤0.01**	16	-0.36	0.17
**EPO vs. LBP**	15	0.21	0.45	27	0.09	0.66	9	0.28	0.46
**EPO vs. Ferritin**	16	0.05	0.86	31	-0.22	0.23	12	-0.60	**≤0.05**
**EPO vs. sCD25**	16	0.25	0.36	31	-0.05	0.78	12	-0.13	0.69
**EPO vs. TNF-α**	15	0.25	0.38	31	0.35	0.05	12	-0.25	0.42
**EPO vs. IL-8**	16	-0.04	0.90	31	0.21	0.25	12	0.19	0.56

**EPO vs. IL-6**	16	-0.03	0.91	27	0.25	0.20	12	-0.51	0.10
**EPO vs. IL-1β**	16	-0.13	0.63	31	0.25	0.18	12	0.11	0.74
**EPO vs. IL-10**	16	0.54	**≤0.05**	31	0.34	0.06	12	0.22	0.50
**LBP vs. Ferritin**	24	0.17	0.43	31	-0.18	0.32	9	-0.25	0.52
**LBP vs. sCD25**	25	0.02	0.91	31	0.35	0.06	9	-0.12	0.78
**LBP vs. TNF-α**	22	-0.53	0.01	31	0.15	0.42	9	0.45	0.23
**LBP vs. IL-8**	24	0.04	0.85	31	0.24	0.20	9	-0.25	0.52
**LBP vs. IL-6**	22	0.07	0.77	29	-0.12	0.54	9	-0.46	0.21
**LBP vs. IL-1β**	25	0.05	0.81	31	-0.08	0.69	9	0.62	0.09
**LBP vs. IL-10**	24	-0.46	**≤0.05**	32	0.10	0.58	9	-0.52	0.16
**Ferritin vs. sCD25**	25	0.19	0.34	34	0.21	0.23	20	0.11	0.63
**Ferritin vs. TNF-α**	22	0.00	0.99	34	-0.26	0.13	16	0.13	0.63
**Ferritin vs. IL-8**	24	-0.22	0.30	34	-0.22	0.21	16	-0.31	0.24
**Ferritin vs. IL-6**	22	0.15	0.51	29	-0.40	0.03	16	0.05	0.86
**Ferritin vs. IL-1β**	25	-0.02	0.92	34	-0.22	0.22	20	0.04	0.86
**Ferritin vs. IL-10**	24	0.23	0.29	35	-0.09	0.62	18	0.07	0.79

Table S2: Correlation analysis of selected CSF cytokine concentrations in AF, SZ, and SAH. r indicates the spearman correlation coefficient. Statistically significant p values (p≤0.05) are highlighted. In contrast to plasma, CSF cytokines show more often negative correlations, such as most of the correlations involving S100B. Interestingly, positive correlations with S100B are restricted to IL-1β, IL-6, and IL-8, exclusively in AF patients (and SAH patients regarding IL-8). A strong negative correlation between IL-10 and IL-8 occurred in AF patients, with a similar trend in SZ patients. SAH patient-derived CSF was restricted to analysis regarding S100B and IL-8.

	**AF**			**SZ**			**SAH**		
	**Size**	**r**	**P value**	**Size**	**r**	**P value**	**Size**	**r**	**P value**
**TNF-α vs. sCD25**	6	-0.29	0.58	5	0.3	0.68	n. d.	n. d.	n. d.
**IL-8 vs. sCD25**	6	0.12	0.84	5	0.5	0.45	n. d.	n. d.	n. d.
**IL-6 vs. sCD25**	6	-0.15	0.83	4	0	1.08	n. d.	n. d.	n. d.
**IL-1β vs. sCD25**	7	-0.41	0.36	5	-0.1	0.95	n. d.	n. d.	n. d.

**IL-10 vs. sCD25**	7	-0.07	0.89	5	0.1	0.95	n. d.	n. d.	n. d.
**S100B vs. sCD25**	4	-0.32	0.67	5	-0.72	0.17	n. d.	n. d.	n. d.
**IL-8 vs. TNF-α**	7	-0.68	0.11	8	-0.62	0.11	n. d.	n. d.	n. d.
**IL-6 vs. TNF-α**	5	-0.10	0.95	6	0.49	0.36	n. d.	n. d.	n. d.
**IL-1β vs. TNF-α**	8	0.79	**≤0.05**	8	0.57	0.15	n. d.	n. d.	n. d.
**IL-10 vs. TNF-α**	8	0.51	0.20	8	0.55	0.17	n. d.	n. d.	n. d.
**S100B vs. TNF-α**	4	-1.00	0.08	7	-0.67	0.12	n. d.	n. d.	n. d.
**IL-6 vs. IL-8**	5	0.30	0.68	6	0.03	1.00	n. d.	n. d.	n. d.
**IL-1β vs. IL-8**	10	-0.21	0.55	11	-0.14	0.69	n. d.	n. d.	n. d.
**IL-10 vs. IL-8**	8	-0.90	**≤0.01**	8	-0.38	0.36	n. d.	n. d.	n. d.
**S100B vs. IL-8**	7	0.64	0.14	9	-0.24	0.54	5	0.50	0.45
**IL-1β vs. IL-6**	6	0.41	0.43	6	0.71	0.14	n. d.	n. d.	n. d.
**IL-10 vs. IL-6**	6	-0.14	0.80	6	0.26	0.66	n. d.	n. d.	n. d.
**S100B vs. IL-6**	3	0.50	1.00	6	-0.03	0.98	n. d.	n. d.	n. d.
**IL-10 vs. IL-1β**	9	0.48	0.19	8	-0.12	0.79	n. d.	n. d.	n. d.
**S100B vs. IL-1β**	7	0.25	0.59	9	-0.20	0.61	n. d.	n. d.	n. d.
**S100B vs. IL-10**	5	-0.70	0.23	7	-0.67	0.12	n. d.	n. d.	n. d.
**EPO vs. LBP**	6	-0.46	0.39	3	-0.50	1.00	n. d.	n. d.	n. d.
**EPO vs. Ferritin**	6	0.70	0.14	3	0.50	1.00	n. d.	n. d.	n. d.
**EPO vs. sCD25**	6	-0.12	0.82	3	0.50	1.00	n. d.	n. d.	n. d.
**EPO vs. TNF-α**	5	0.90	0.08	3	0.50	1.00	n. d.	n. d.	n. d.
**EPO vs. IL-8**	6	-0.99	**≤0.05**	3	-1.00	0.33	n. d.	n. d.	n. d.
**EPO vs. IL-6**	5	-0.46	0.43	-	-	-	n. d.	n. d.	n. d.
**EPO vs. IL-1β**	6	0.60	0.22	3	-0.50	1.00	n. d.	n. d.	n. d.
**EPO vs. IL-10**	6	0.99	**≤0.05**	3	1.00	0.33	n. d.	n. d.	n. d.
**EPO vs. S100B**	5	-0.95	0.17	3	-0.87	0.67	n. d.	n. d.	n. d.
**LBP vs. Ferritin**	7	0.14	0.78	5	-0.40	0.52	n. d.	n. d.	n. d.

**LBP vs. sCD25**	7	0.22	0.64	4	0.20	0.92	n. d.	n. d.	n. d.
**LBP vs. TNF-α**	6	0.03	1.00	5	-0.60	0.35	n. d.	n. d.	n. d.
**LBP vs. IL-8**	6	0.43	0.42	5	0.80	0.13	n. d.	n. d.	n. d.
**LBP vs. IL-6**	6	0.14	0.80	4	0.20	0.92	n. d.	n. d.	n. d.
**LBP vs. IL-1β**	7	0.23	0.61	5	-0.60	0.35	n. d.	n. d.	n. d.
**LBP vs. IL-10**	7	-0.32	0.50	5	-0.50	0.45	n. d.	n. d.	n. d.
**LBP vs. S100B**	4	0.40	0.75	5	0.36	0.57	n. d.	n. d.	n. d.
**Ferritin vs. sCD25**	7	-0.19	0.72	5	0.80	0.13	n. d.	n. d.	n. d.
**Ferritin vs. TNF-α**	8	0.91	**≤0.05**	8	0.71	0.06	n. d.	n. d.	n. d.
**Ferritin vs. IL-8**	8	-0.71	0.06	8	-0.29	0.51	n. d.	n. d.	n. d.
**Ferritin vs. IL-6**	6	0.37	0.50	6	-0.03	1.00	n. d.	n. d.	n. d.
**Ferritin vs. IL-1β**	9	0.78	**≤0.05**	8	0.50	0.22	n. d.	n. d.	n. d.
**Ferritin vs. IL-10**	9	0.56	0.12	8	0.12	0.79	n. d.	n. d.	n. d.
**Ferritin vs. S100B**	5	-0.40	0.52	7	-0.93	**≤0.01**	n. d.	n. d.	n. d.
